# Real-world effectiveness and safety of sofosbuvir and ledipasvir with or without ribavirin for patients with hepatitis C virus genotype 1 infection in Taiwan

**DOI:** 10.1371/journal.pone.0209299

**Published:** 2018-12-21

**Authors:** Chen-Hua Liu, Chun-Jen Liu, Tung-Hung Su, Hung-Chih Yang, Chun-Ming Hong, Tai-Chung Tseng, Pei-Jer Chen, Ding-Shinn Chen, Jia-Horng Kao

**Affiliations:** 1 Department of Internal Medicine, National Taiwan University Hospital, Taipei, Taiwan; 2 Hepatitis Research Center, National Taiwan University Hospital, Taipei, Taiwan; 3 Department of Internal Medicine, National Taiwan University Hospital, Yun-Lin Branch, Douliou, Taiwan; 4 Graduate Institute of Clinical Medicine, National Taiwan University College of Medicine, Taipei, Taiwan; 5 Department of Microbiology, National Taiwan University College of Medicine, Taipei, Taiwan; 6 Genomics Research Center, Academia Sinica, Taipei, Taiwan; Nihon University School of Medicine, JAPAN

## Abstract

**Background:**

The real-world data for the effectiveness and safety of sofosbuvir/ledipasvir (SOF/LDV) with or without ribavirin (RBV) in patients with hepatitis C virus genotype 1 (HCV-1) infection remain limited in Taiwan.

**Methods:**

A total of 273 chronic HCV-1 patients receiving 8, 12, or 24 weeks of SOF/LDV with or without RBV were enrolled. The sustained virologic response rate at week 12 off-therapy (SVR_12_) by evaluable population (EP) and per-protocol population (PP) were assessed for effectiveness. The treatment discontinuation rate due to adverse events (AEs) and serious AE rate were assessed for safety. Baseline patient characteristics and on-treatment HCV viral kinetics associated with SVR_12_ were analyzed.

**Results:**

The SVR_12_ rates by EP and PP analyses were 96.7% (95% confidence interval [CI]: 93.9%-98.3%) and 97.5% (95% CI: 94.8%-98.8%), respectively. The rates of treatment discontinuation due to AE and serious AE were 0.4% and 4.4%, respectively. Seven patients with true virologic failure were relapsers. In 2 patients who were lost-to follow-up, one expired at treatment week 3 due to pneumonia which was considered not related to treatment, and one declined follow-up at off-therapy week 4. The SVR_12_ rates were comparable in terms of baseline patient characteristics and viral decline at week 4 of treatment.

**Conclusions:**

SOF/LDV with or without RBV for 8–24 weeks is well tolerated and achieves a high SVR_12_ rate in patients with HCV-1 infection in Taiwan.

## Introduction

Hepatitis C virus (HCV) infection is a challenging health problem which affects approximately 71.1 million people worldwide [1]. Over a period of 20–30 years, about 20% of chronic HCV-infected patients will evolve to cirrhosis which may progress to hepatic decompensation and hepatocellular carcinoma (HCC) [2,3]. Apart from the liver-related morbidity and mortality, HCV infection may also induce extra-hepatic manifestations which adversely affects the patients’ health outcome and quality of life [4]. On the other hand, the prognosis is improved once patients achieve sustained virologic response (SVR) following anti-HCV agents [5–8]. Currently, HCV genotype 1 (HCV-1) infection is predominant around the world [9]. Compared to patients with non-HCV-1 infection, those with HCV-1 infection have an increased risk of cirrhosis and HCC [10,11]. Therefore, an effective and safe HCV treatment strategy, particularly for patients with HCV-1infection, is mandatory.

The introduction of interferon (IFN)-free direct acting antiviral agents (DAAs) has revolutionized the care of HCV infection. Sofosbuvir (SOF) is a pyrimidine nucleotide analogue that inhibits the HCV non-structural protein 5B (NS5B) ribonucleic acid (RNA)-dependent RNA polymerase. After intra-hepatic metabolism, the active uridine triphosphate form is incorporated to HCV RNA by NS5B polymerase and acts as the chain terminator [12]. Clinically, SOF is administered once-daily with pangenotypic potency, excellent tolerability, high genetic barriers to drug resistance, and few potential drug-drug interactions (DDIs). Currently, SOF can be used with ledipasvir (LDV) as a formula of fixed-dose combination which is active against HCV-1, 4, 5 or 6 infection. The efficacy and safety of SOF/LDV with or without RBV for 8–24 weeks for ordinary HCV-1 patients are excellent in phase III trials [13–16]. Furthermore, the therapeutic profiles remain excellent among patients with human immunodeficiency virus (HIV) coinfection, decompensated cirrhosis, or organ transplantation [17–21]. Therefore, SOF/LDV-based regimens for HCV-1 patients are appealing to most health care providers.

Regarding to the real-world effectiveness and safety of SOF/LDV with or without RBV for HCV-1 patients, data from Western and Eastern countries showed that the SVR rates ranged from 92%-98% and most patients tolerated the treatment well [22–26]. On the basis of these encouraging results, we aimed to evaluate the real-world performance of SOF/LDV with or without RBV for HCV-1 patients in Taiwan.

## Materials and methods

### Patients

Between April 2015 and August 2017, HCV-1 infected patients who received SOF/LDV for 8, 12 or 24 weeks with or without ribavirin (RBV) were retrospectively enrolled at the National Taiwan University Hospital (NTUH) and NTUH Yun-Lin Branch. All patients were aged ≥ 20 years and had chronic HCV infection, defined as detectable HCV antibody (anti-HCV; Abbott HCV EIA 2.0, Abbott Laboratories, Abbott Park, Illinois, USA) and quantifiable serum HCV RNA (Cobas TaqMan HCV Test v2.0, Roche Diagnostics GmbH, Mannheim, Germany, lower limit of detection [LLOD]: 15 IU/mL) for ≥ 6 months. Patients who had non-HCV-1 infection, had prior DAA exposure, had active HCC, had estimated glomerular filtration rate (eGFR) < 30 mL/min/1.73m^2^, received treatment regimens outside the guideline recommendation, or refused to provide written informed consent were excluded from the study [27–29]. The study was approved by the NTUH Research Ethics Committee (201205058RIC) and was conducted in accordance with the principles of Declaration of Helsinki and the International Conference on Harmonization for Good Clinical Practice. All patients provided written informed consent before the study.

### Study design

Baseline patient demographics, hemogram, serum biochemical data (albumin, total bilirubin, aspartate aminotransferase [AST], alanine aminotransferase [ALT], creatinine, eGFR, as calculated by modification of diet in renal disease (MDRD) equation, anti-HCV, hepatitis B virus (HBV) surface antigen (Abbott Architect HBsAg qualitative assay, Abbott Laboratories, Abbott Park, Illinois, USA), HCV RNA, HCV genotype (Abbott RealTi*me* HCV Genotype II, Abbott Laboratories, Abbott Park, Illinois, USA) and anti-HIV (Abbott Architect HIV Ag/Ab Combo, Abbott Laboratories, Abbott Park, Illinois, USA) were collected [30]. The status of cirrhosis was determined by liver biopsy, clinical signs of portal hypertension, imaging studies, AST-to-platelet ratio index (ARPI) with a cutoff value of > 2.0 or liver stiffness measurement (LSM, FibroScan, Echosens, Paris, France) with a cutoff value of > 12.5 kPa when appropriate [31,32]. The stage of cirrhosis was graded by Child-Pugh score. Baseline serum HBV DNA (Cobas AmpliPrep/Cobas Taqman HBV test v.2.0, Roche Diagnostics GmbH, Mannheim, Germany, LLOD: 20 IU/mL) or HIV RNA (Cobas AmpliPrep/Cobas Taqman HIV-1 test v.2.0, Roche Diagnostics GmbH, Mannheim, Germany, LLOD: 20 copies/mL) level was determined for patients with HBV or HIV coinfection [33].

Patients received fixed-dose combination of SOF/LDV (400mg/90mg, Harvoni, Gilead Sciences, Carrigtohill, Co. Cork, Ireland) 1 tablet per day for 8, 12 or 24 weeks. Treatment-naïve, non-cirrhotic patients with baseline HCV RNA level < 6,000,000 IU/mL can receive 8 or 12 weeks of SOF/LDV treatment. Patients with compensated cirrhosis (Child-Pugh A) can receive 12 weeks of SOF/LDV with or without weight-based RBV (Robatrol, 200 mg capsule, Genovate Biotechnology Co. Ltd., Hsinchu, Taiwan; 1,200 mg per day if the body weight ≥ 75 kg; 1,000 mg per day if the body weight < 75 kg) or 24 weeks of SOF/LDV at the discretion of the physicians. Patients who had decompensated cirrhosis (Child-Pugh B or C) or had undergone liver transplantation can receive 12 weeks of SOF/LDV with weight-based RBV or 24 weeks of SOF/LDV. For patients with baseline eGFR between 30–50 mL/min/1.73m^2^, the RBV was adjusted to 200 mg/400 mg per day at alternative dosage.

### Effectiveness

Patients received on-treatment serum HCV RNA monitoring at week 4 and at the end of treatment (EOT). Furthermore, they received off-therapy serum HCV RNA testing at week 12 to assess SVR_12_. Patients were considered failure to achieve SVR_12_ if they lacked SVR_12_ data. We adopted two different endpoints for effectiveness: the evaluable population (EP) which assessed the SVR_12_ for patients who received at least one dosage of treatment, and the per-protocol population (PP) which assessed the SVR_12_ by excluding non-SVR_12_ patients due to non-virologic failure.

### Safety

The rate of treatment completion was assessed for all patients. The reasons for patients who prematurely discontinued treatment or were lost-to follow-up were assessed through the chart review. The on-treatment constitutional and laboratory adverse events (AEs), and serious AEs were also evaluated. In patients who were seropositive for HBsAg, serum HBV DNA levels were evaluated after the initiation of DAA treatment. HBV reactivation was defined as the presence of HBV DNA level ≥ LLOD in patients with baseline HBV DNA level < LLOD, or increase of HBV DNA level > 1 log_10_ IU/mL in patients with baseline HBV DNA level ≥ LLOD [34]. HBV-associated hepatitis was defined as HBV reactivation and hepatitis flare presenting with ALT increase ≥ 3 times baseline and > 100 U/L [35].

### Statistical analysis

All analyses were performed using Statistical Program for Social Sciences (SPSS Statistics Version 23.0, IBM Corp., Armonk, New York, USA). The baseline characteristics were shown in median (range) and numbers (percentages) when appropriate. The rates of antiviral response were shown in numbers (percentages) with 95% confidence interval (CI) and the AE rates were shown in numbers (percentages). The stratified analysis of SVR_12_ by EP analysis for baseline characteristics and week 4 viral decline were assessed and shown in percentages with 95% CI.

## Results

### Patient characteristics

**Fig 1:**
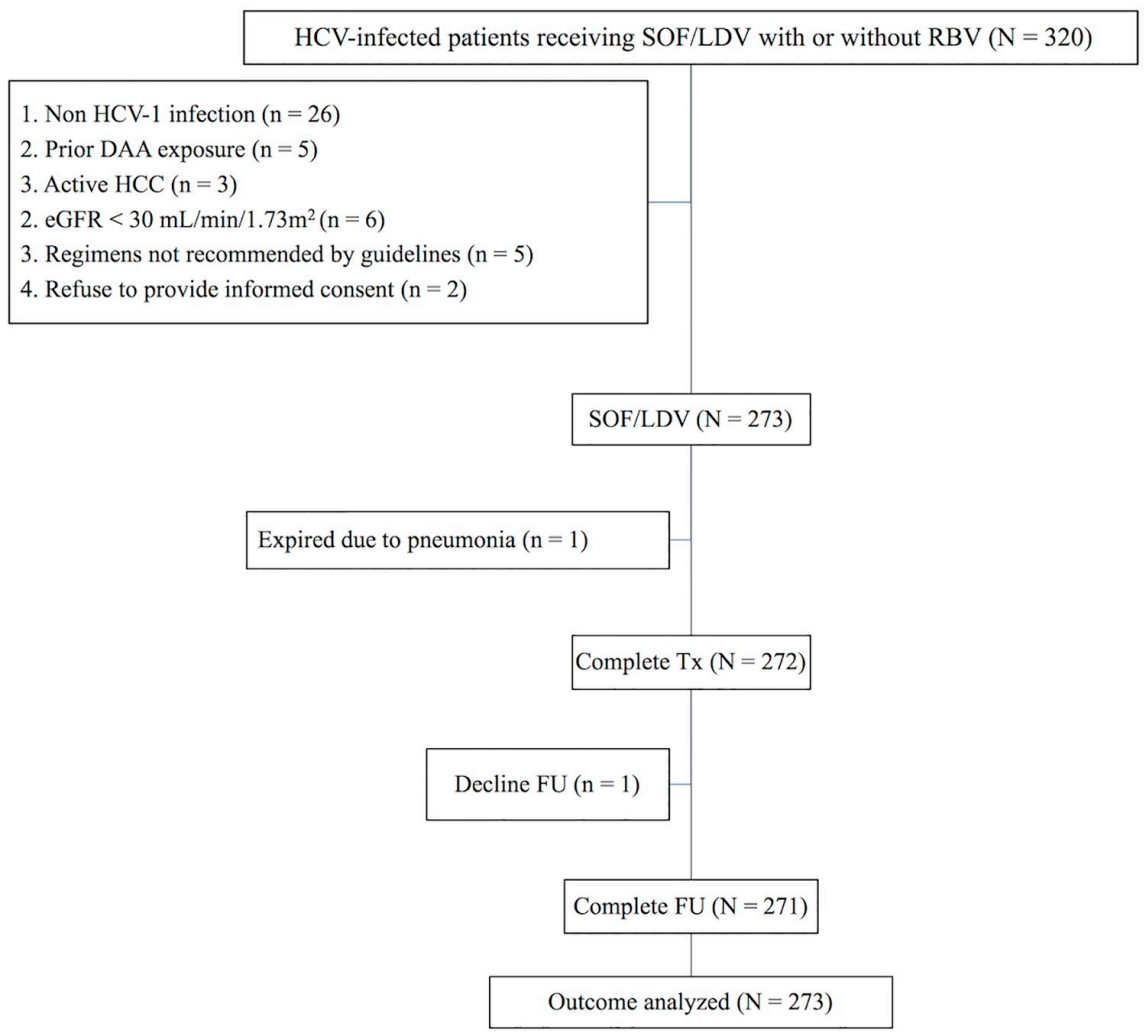
Study flow.

Of 320 HCV-infected patients receiving SOF/LDV with or without RBV, 47 were excluded from the study because of non-HCV-1 infection, prior DAA exposure, active HCC, eGFR < 30 mL/min/1.73m^2^, receiving antiviral regimens not recommended by guidelines, or refusal to provide informed consent. The remaining 273 patients were eligible for the analysis (Fig 1). Table 1 shows the baseline patient characteristics. One hundred twenty-seven (46.5%) patients were male and 182 (66.7%) were treatment-naïve. Nine (3.3%) and 13 (4.8%) patients had HBV and HIV coinfection, respectively. Twenty-six (9.5%) and 13 (4.8%) patients underwent liver and renal transplantation, respectively. Among the 88 (32.2%) patients receiving RBV, all received treatment for 12 weeks, including 25 who underwent liver transplantation (one with decompensated cirrhosis), 25 with decompensated cirrhosis (one who underwent liver transplantation), and 39 with compensated cirrhosis (37 with prior IFN-based treatment). All patients who underwent renal transplantation were treatment-naïve and received SOF/LDV for 12 weeks. One decompensated cirrhotic, one treatment-naïve compensated cirrhotic, and one liver transplantation patients received SOF/LDV for 24 weeks. With regard to HCV subgenotype distribution, 21 (7.7%), 242 (88.6%) and 10 (3.7) patients had HCV-1a, HCV-1b, and unsubtypable HCV-1 infections. One hundred thirty-eight (50.5%) patients were non-cirrhotic. Among the 135 cirrhotic patients, 109 (80.7) and 26 (19.3%) of them had compensated and decompensated cirrhosis, respectively. Seven (77.8%) and 12 (92.3%) patients with HBV or HIV coinfection had baseline undetectable serum HBV DNA or HIV RNA.

**Table 1:** Baseline patient characteristics.

Characteristics*	Patient (N = 273)
**Age, year, median (range)**	64 (29–86)
**Age ≥ 60 years**	188 (68.9)
**Male**	127 (46.5)
**Treatment-naive**	182 (66.7)
**HBV coinfection**	9 (3.3)
**HIV coinfection**	13 (4.8)
**Prior history of HCC**	57 (20.9)
**Liver transplantation**	26 (9.5)
**Renal transplantation**	13 (4.8)
**Treatment duration, week**	
8 weeks	5 (1.8)
12 weeks	265 (97.1)
24 weeks	3 (1.1)
**RBV usage**	88 (32.2)
**Hemoglobin, g/dL, median (range)**	13.5 (7.7–17.6)
**White cell count, 10**^**9**^ **cells/L, median (range)**	4.9 (1.9–12.5)
**Platelet count, 10**^**9**^ **cells/L, median (range)**	136 (33–433)
**Albumin, g/dL, median (range)**	4.1 (2.1–5.0)
**Total bilirubin, mg/dL, median (range)**	0.8 (0.3–8.8)
**AST, ULN, median (range)**	1.7 (0.3–8.5)
**ALT, ULN, median (range)**	1.7 (0.3–10.6)
**Creatinine, mg/dL, median (range)**	0.8 (0.4–2.1)
**eGFR, mL/min/1.73m**^**2**^, **median (range)** †	84 (32–186)
**eGFR < 60 mL/min/1.73m**^**2**^ †	52 (19.0)
**HCV RNA, log**_**10**_ **IU/mL, median (range)**	6.17 (2.85–7.69)
**HCV RNA > 6,000,000 IU/mL**	48 (17.6)
**HCV genotype**	
1a	21 (7.7)
1b	242 (88.6)
1‡	10 (3.7)
**Cirrhosis**	
Absent	138 (50.5)
Present	135 (49.5)
Child-Pugh A	109 (80.7)
Child-Pugh B and C	26 (19.3)

HBV: hepatitis B virus; HIV: human immunodeficiency virus; HCC: hepatocellular carcinoma; RBV: ribavirin; AST: aspartate aminotransferase; ALT: alanine aminotransferase; ULN: upper limit of normal; eGFR: estimated glomerular filtration rate.

* Values are numbers (percentages) unless otherwise indicated.

^†^ eGFR was calculated by MDRD equation.

^‡^ Failed subtyping for major genotyping.

### Effectiveness

Of the 272 patients with available HCV RNA data at week 4 of treatment, 218 (80.2%) of them had undetectable serum HCV RNA. All 272 (100%) patients with available HCV RNA data had undetectable HCV RNA at EOT. One Child-Pugh C cirrhotic patient receiving SOF/LDV with RBV died at treatment week 3 did not receive HCV RNA testing at week 4 and EOT. The overall SVR_12_ rates were 96.7% (264 of 273 patients; 95% CI: 93.9%-98.3%) by EP analysis, and 97.5% (264 of 271 patients; 95% CI: 94.8%-98.8%) by PP analysis (Table 2).

**Table 2:** Virologic responses.

HCV RNA < LLOD	Patient (N = 273)	
	n/N (%)	95% CI
**During treatment**		
Week 4*	218/272 (80.2)	75.0–84.5
EOT*	272/272 (100)	98.6–100
**After treatment**		
SVR_12_ (EP)†	264/273 (96.7)	93.9–98.3
SVR_12_ (PP)‡	264/271 (97.5)	94.8–98.8
**Reason for non-SVR**_**12**_, **n**		
Relapse	7	
Lost to follow-up	2	
During treatment	1	
After treatment	1	

LLOD: lower limit of detection; EOT: end-of-treatment; EP: evaluate population; PP: per-protocol population; CI: confidence interval.

* One patient who expired at treatment week 3 did not have week 4 and EOT HCV RNA data.

^†^ Patients who received at least one dosage of treatment were included in the analysis.

^‡^ Patients with non-virologic failure were excluded from the analysis.

Among patients who failed to achieved SVR_12_, 7 (2.6%) were relapsers and 2 (0.7%) were lost to follow-up. Among the relapser, 5 (71.4%) were female, 6 (85.7%) were treatment-naïve, 4 (57.1%) had cirrhosis and all received SOF/LDV with or without RBV for 12 weeks. In 2 patients who were lost to follow-up, one died of pneumonia at treatment week 3, and the other one declined follow-up at post-treatment week 4 (S1 Table).

### Stratified analysis of patient characteristics predictive of SVR_12_

Table 3 shows the stratified SVR_12_ rates of SOF/LDV with or without RBV by baseline characteristics and week 4 treatment response. The SVR_12_ rates were comparable with regard to age at a cut-off value of 60 years, sex, prior IFN exposure, HBV or HIV coinfection, liver or renal transplantation, scheduled treatment duration, use of RBV, eGFR at a cut-off value of 60 mL/min/1.73m^2^, baseline HCV viral load at a cut-off value of 6,000,000 IU/mL, HCV genotype, cirrhosis and week 4 viral decline. The SVR_12_ rates for patients with HCV-1a, HCV-1b, and unsubtypable HCV-1 infections were 90.5% (95% CI: 71.1%-97.4%), 97.9% (95% CI: 95.3%-99.1%), and 80.0% (95% CI: 49.0%-94.3%), respectively. Among the cirrhotic patients, the SVR_12_ rates for those with compensated and decompensated cirrhosis were 97.3% (95% CI: 92.2%-99.1%) and 88.5% (95% CI: 71.0%-96.0%), respectively.

**Table 3:** SVR_12_ according to baseline patient characteristics and on-treatment HCV viral decline.

Characteristics	Patient (N = 273)		
	Patient No.	SVR_12_ (%)	95% CI
**Age, years**			
< 60	85	94.1	87.0–97.5
≥ 60	188	97.9	94.7–99.2
**Sex**			
Female	146	96.6	92.2–98.5
Male	127	96.9	92.2–98.8
**Treatment experience**			
Naïve	182	96.7	93.0–98.5
Experienced	91	96.7	90.8–98.9
**HBV coinfection**			
Absent	264	96.6	93.7–98.2
Present	9	100	70.1–100
**HIV coinfection**			
Absent	260	96.5	93.6–98.2
Present	13	100	77.2–100
**Prior HCC history**			
Absent	216	96.8	93.5–98.4
Present	57	96.5	88.1–99.0
**Liver transplantation**			
No	247	96.4	93.2–98.1
Yes	26	100	87.1–100
**Renal transplantation**			
No	260	96.5	93.6–98.2
Yes	13	100	77.2–100
**Treatment duration, week**			
8	5	100	56.6–100
12	265	96.6	93.7–98.2
24	3	100	43.9–100
**RBV usage**			
No	185	97.3	93.8–98.8
Yes	88	95.5	88.9–98.2
**eGFR, mL/min/1.73m**^**2**^			
< 60	52	98.1	89.9–99.7
≥ 60	221	96.4	93.0–98.2
**HCV RNA, IU/mL**			
< 6,000,000	225	96.4	93.1–98.2
≥ 6,000,000	48	97.9	89.1–99.6
**HCV genotype**			
1a	21	90.5	71.1–97.4
1b	242	97.9	95.3–99.1
1	10	80.0	49.0–94.3
**Cirrhosis**			
Absent	138	97.8	93.8–99.3
Present	135	95.6	90.7–98.0
Child-Pugh A	109	97.3	92.2–99.1
Child-Pugh B and C	26	88.5	71.0–96.0
Week 4 HCV RNA < LLOD*			
No	54	94.4	84.9–98.1
Yes	218	97.7	94.8–99.0

NA: not assessed.

* One patient who expired at treatment week 3 did not have week 4 HCV RNA data.

In patients who were treatment-naïve, non-cirrhotic and had baseline HCV RNA level < 6,000,000 IU/mL, the SVR_12_ rates were 100% (95% CI: 56.6%-100%) and 98.7% (95% CI: 92.8%-99.8%) for patients receiving 8 and 12 weeks of SOF/LDV. The SVR_12_ rates of SOF/LDV for 12 weeks in compensated cirrhotic patients who were treatment-naïve and treatment-experienced were 96.5% (95% CI: 88.1%-99.0%) and 100% (95% CI: 61.0%-100%), respectively. Furthermore, the SVR_12_ rates of SOF/LDV with RBV for 12 weeks in compensated cirrhotic patients who were treatment-naïve and treatment-experienced were 100% (95% CI: 61.0%-100%) and 97.4% (95% CI: 86.8%-99.6%), respectively. In patients with decompensated cirrhosis, the SVR_12_ rates of SOF/LDV with RBV for 12 weeks and SOF/LDV for 24 weeks were 88% (95% CI: 70.0%-95.8%) and 100% (95% CI: 20.7%-100%), respectively (Table 4).

**Table 4:** SVR_12_ in patients of specific interest.

Patients of specific interest*	Patient No.	SVR_12_ (%)	95% CI
**Treatment-naïve, non-cirrhotic, & baseline HCV RNA level < 6,000,000 IU/mL**			
SOF/LDV, 8 weeks	5	100	56.6–100
SOF/LDV, 12 weeks	75	98.7	92.8–99.8
**Treatment-naïve, Child-Pugh A cirrhotic**			
SOF/LDV, 12 weeks	57	96.5	88.1–99.0
SOF/LDV with RBV, 12 weeks	6	100	61.0–100
SOF/LDV, 24 weeks	1	100	20.7–5100
**Treatment-experienced, Child-Pugh A cirrhotic**			
SOF/LDV, 12 weeks	6	100	61.0–100
SOF/LDV with RBV, 12 weeks	39	97.4	86.8–99.6
**Child-Pugh B or C cirrhotic**			
SOF/LDV with RBV, 12 weeks	25	88	70.0–95.8
SOF/LDV, 24 weeks	1	100	20.7–100

* Patients receiving liver or renal transplantation were not included in the analysis.

### Safety

Two hundred seventy-two (99.6%) patients completed the scheduled treatment. One treatment-naïve Child-Pugh C cirrhotic patients receiving SOF/LDV with RBV died at treatment week 3 due to pneumonia, which was not related to treatment. Twelve (4.4%) patients experienced on-treatment serious AEs, and none were considered related to DAA treatment. The rates and severity of hematological and hepatic AEs were generally low and mild in grade. The common AEs with event rates ≥ 10% included fatigue (27.1%), headache (20.5%), nausea (17.9%) and insomnia (13.9%) (Table 5). The rates for serious AE, fatigue, hemoglobin level < 10 g/dL, and elevated total bilirubin level were higher in patients with decompensated cirrhosis than those with no cirrhosis and with compensated cirrhosis. Among the 9 patients with HBV coinfection, 2 (22.2%) experienced HBV reactivation after treatment, but none had HBV-associated hepatitis that needed anti-HBV treatment.

**Table 5:** Safety summary.

Variable, n (%)	All patient (N = 273)	No cirrhosis (n = 138)	Child-Pugh A cirrhosis (n = 109)	Child-Pugh B/C cirrhosis (n = 26)
**Serious adverse event**	12 (4.4)	1 (0.7)	5 (4.6)	6 (23.1)
Pneumonia	1 (0.4)	0 (0)	0 (0)	1 (3.8)
Spontaneous bacterial peritonitis	3 (1.1)	0 (0)	0 (0)	3 (11.5)
Variceal bleeding	2 (0.7)	0 (0)	1 (0.9)	1 (3.8)
Hepatocellular carcinoma	4 (1.5)	0 (0)	3 (2.8)	1 (3.8)
Herpes zoster	1 (0.4)	0 (0)	1 (0.9)	0 (0)
Duodenal ulcer bleeding	1 (0.4)	1 (0.7)	0 (0)	0 (0)
Discontinuation due to adverse event*	1 (0.4)	0 (0)	0 (0)	1 (3.8)
Death*	1 (0.4)	0 (0)	0 (0)	1 (3.8)
**Adverse event in ≥ 10% of patients**				
Fatigue	74 (27.1)	30 (21.7)	32 (29.4)	12 (46.2)
Headache	56 (20.5)	28 (20.3)	22 (20.2)	6 (23.1)
Nausea	49 (17.9)	22 (15.9)	21 (19.3)	6 (23.1)
Insomnia	38 (13.9)	18 (13.0)	15 (13.8)	5 (19.2)
**Laboratory adverse event**				
Hemoglobin				
8.0–10.0 g/dL	21 (7.7)	1 (0.7)	14 (12.8)	6 (23.1)
< 8.0 g/dL	4 (1.5)	0 (0)	3 (2.8)	1 (3.8)
White blood cell count				
2.0–3.0 x 10^9^ cells/L	7 (2.6)	0 (0)	5 (4.6)	2 (7.7)
< 2.0 x 10^9^ cells/L	2 (0.7)	0 (0)	1 (0.9)	1 (3.8)
Platelet count				
50–75 x 10^9^ cells/L	46 (16.8)	3 (2.2)	24 (22.0)	19 (73.1)
< 50 x 10^9^ cells/L	10 (3.7)	0 (0)	4 (3.7)	6 (19.2)
Total bilirubin				
1.5–3.0 x ULN	21 (7.7)	2 (1.4)	11 (10.1)	8 (30.8)
> 3.0 x ULN	9 (3.3)	0 (0)	4 (3.7)	5 (19.2)
ALT				
3–5 x ULN	6 (2.2)	3 (2.2)	2 (1.8)	1 (3.8)
> 5x ULN	2 (0.7)	1 (0.7)	1 (0.9)	0 (0)
eGFR				
15–30 mL/min/1.73m^2^	3 (1.1)	0 (0)	2 (1.8)	1 (3.8)
< 15 mL/min/1.73m^2^	0 (0)	0 (0)	0 (0)	0 (0)

* One patient expired due to pneumonia at treatment week 3, which was considered not related to DAA treatment.

## Discussion

Compared to protease inhibitor (PI)-containing HCV DAA regimens for HCV-1 infection, the PI-free SOF/LDV regimen has lower pill burden, fewer potential drug-drug interactions (DDIs), and can be applied to decompensated cirrhotic patients [36–38]. Therefore, treatment by SOF/LDV with or without RBV is appealing to most health care providers in the management of HCV-1 infection.

Our real-world study which enrolled a heterogeneous group of HCV-1 patients showed that the SVR_12_ rates by EP and PP analyses in patients receiving SOF/LDV with or without RBV for 8–24 weeks were excellent (96.7% and 97.5%, respectively) and were comparable to the response rates in clinical trials and real-world studies [13,14,15,17–20,23–26]. Furthermore, 99.6% of our patients completed the scheduled treatment and 4.4% of them experienced on-treatment serious AEs, which were also comparable to the pooled safety analysis for patients receiving SOF/LDV with or without RBV for 8–24 weeks in ION studies [16]. Only one decompensated cirrhotic patient prematurely discontinued treatment due to pneumonia, which was considered not related to SOF/LDV. The rates of common constitutional AEs, including fatigue, headache, nausea, and insomnia were also in line with the ION reports [16]. However, patients with decompensated cirrhosis tended to have higher risks of serious AE, fatigue, anemia and hyperbilirubinemia than those with no cirrhosis and with compensated cirrhosis, implying that the treating physicians should be alert to the clinical presentations in patients with decompensated cirrhosis to secure the safety profiles [18,19]. Based on the excellent safety and effectiveness in our study, applying SOF/LDV with or without RBV may serve as an ideal regimen for HCV-1 infection.

In terms of patient characteristics, our study showed that the SVR_12_ rates were similar regardless of age, sex, prior treatment experience, HBV or HIV coinfection, prior HCC history, HCV viral load, eGFR level or week 4 viral decline [17,39–41]. The SVR_12_ rate in compensated cirrhotic patients was also comparable to non-cirrhotic patients [13,14]. Furthermore, the SVR_12_ rate in decompensated cirrhotic patients was 88.5% (95% CI: 71.0%-96%) and was comparable to the reports in SOLAR-1 and SOLAR-2 studies [18,19]. Patients with decompensated cirrhosis had lower SVR_12_ rate than patients with no cirrhosis or with compensated cirrhosis, probably due to lower drug delivery, altered drug metabolism, and impaired immune response in these patients [42–44]. Applying velpatasvir (VEL), which exhibits a higher genetic barrier to N55A resistance associated substitutions (RASs) than LDV, in combination with SOF and RBV for 12 weeks, or treating patients following liver transplantation, may improve the clinical outcome in decompensated cirrhotic patients [18,19,32,45]. Although there were no statistical differences, the SVR_12_ rate in patients with HCV-1a infection were numerically lower than that with HCV-1b infection, which may be reasoned by the greater loss of response rates for HCV-1a patients receiving SOF/LDV than for HCV-1b patients in the presence of NS5A RASs [46].

Among our HCV-1 patients receiving liver transplantation, 25 patients were treated by SOF/LDV with RBV for 12 weeks and one were treated by SOF/LDV for 24 weeks. All of them achieved SVR_12_, implying that the effectiveness of SOF/LDV-based therapies remained excellent in this special population [18,19]. In contrast, 13 HCV-1 patients receiving renal transplantation were treated by SOF/LDV for 12 weeks and all achieved SVR_12_, implying that RBV-free SOF/LDV regimen can be applied to patients receiving non-liver solid organ transplantation [20,21,47].

In ION-3 and real-world studies, treatment-naïve, non-cirrhotic HCV-1 patients with baseline HCV RNA < 6,000,000 IU/mL can receive SOF/LDV for 8 weeks without compromising the treatment responses [15,48]. All 5 (100%) patients and 74 of 75 (98.7%) patients who met such criteria achieved SVR_12_ by 8 and 12 weeks of SOF/LDV, respectively. In treatment-naïve, compensated cirrhotic HCV-1 patients, our study showed that adding RBV to SOF/LDV for 12 week or extending SOF/LDV treatment to 24 weeks did not benefit the SVR_12_ rates, compared to SOF/LDV for 12 weeks [49]. In contrast to Western studies, our data were in line with Asian reports indicating that there was no benefit to improve the SVR_12_ rate by adding RBV to SOF/LDV for 12 weeks in treatment-experienced, compensated cirrhotic HCV-1 patients [50,51]. Further studies are needed to explore the potential mechanisms for such discrepancies.

Among the 9 patients with HBV coinfection, the risks of HBV reactivation and the HBV-related hepatitis after DAA treatment were 22.2% and 0%, which were comparable to the report in a meta-analysis enrolling 242 HBV-coinfected patients [52]. Although there were no apparent clinical events related to HBV reactivation in our study, watchful surveillance of HBV activity is still needed to detect and treat potential complications related to HBV reactivation at the earliest stage.

Although we confirmed that SOF/LDV with or without RBV had excellent safety and effectiveness for HCV-1 patients in Taiwan, several limitations existed in our study. First, the numbers of patients receiving SOF/LDV for 8 or 24 weeks were small and more data are needed to confirm the overall performance in patients of specific interests. Second, HCV-6 patients may potentially be misclassified to unsubtypable HCV-1 patients by Abbott RealTi*me* HCV Genotype II testing, which might affect the SVR_12_ rate in our study [53]. Third, we did not evaluate the effects baseline NS5A RASs on the treatment responses in our patients, particularly for HCV-1a patients.

In summary, SOF/LDV with or without RBV for 8–24 weeks is well tolerated and achieves a high SVR_12_ rate in HCV-1 infection, which may improve the care of such patients in Taiwan.

## Supporting information

S1 Table Summary of patients who did not achieve SVR_12_. (DOCX)
