# Supplementary material for: Real-world effectiveness and safety of sofosbuvir and ledipasvir with or without ribavirin for patients with hepatitis C virus genotype 1 infection in Taiwan
Source: PLoS One. 2018 Dec 21;13(12):e0209299. doi: 10.1371/journal.pone.0209299 (PMC6303025; doi:10.1371/journal.pone.0209299)
Supplement: S1 Table — (DOCX) [file pone.0209299.s001.docx]

**S1 Table. Summary of patients who did not achieve SVR_12_**

| **Patient No.** | **Age** | **Sex** | **Tx experience** | **HCV RNA, log_10_ IU/mL** | **HCV GT** | **Cirrhosis** | **Child-Pugh** | **Tx regimen** | **Scheduled Tx, week** | **Actual Tx, week** | **Others** |
| --- | --- | --- | --- | --- | --- | --- | --- | --- | --- | --- | --- |
| **Relapse** |  |  |  |  |  |  |  |  |  |  |  |
| 1 | 53 | M | Naïve | 5.89 | 1a | Present | A | SOF/LDV | 12 | 12 | - |
| 2 | 58 | F | Naïve | 7.09 | 1b | Present | A | SOF/LDV | 12 | 12 | - |
| 3 | 67 | M | Experienced | 5.45 | 1a | Absent | - | SOF/LDV | 12 | 12 | - |
| 4 | 46 | F | Naïve | 6.46 | 1b | Absent | - | SOF/LDV | 12 | 12 | - |
| 5 | 64 | F | Naïve | 6.41 | 1 | Present | B | SOF/LDV/RBV | 12 | 12 | - |
| 6 | 70 | F | Naïve | 6.32 | 1b | Present | B | SOF/LDV/RBV | 12 | 12 | - |
| 7 | 54 | F | Naïve | 6.64 | 1b | Absent | - | SOF/LDV | 12 | 12 | - |
| **LTFU** |  |  |  |  |  |  |  |  |  |  |  |
| 1 | 68 | M | Naïve | 6.63 | 1b | Present | C | SOF/LDV/RBV | 12 | 2 | Expired due to pneumonia at TW3 |
| 2 | 38 | M | Experienced | 6.59 | 1 | Present | A | SOF/LDV/RBV | 12 | 12 | Declined FU at PTW4 |

Tx: treatment, GT: genotype, FU: follow-up, PTW: post-treatment week, TW: treatment week
